# Discussions about physical activity in general practice: analysis of video-recorded consultations

**DOI:** 10.3399/BJGP.2024.0166

**Published:** 2025-02-11

**Authors:** Adam Grice, Amy S Izon, Nada F Khan, Robbie Foy, Rebecca J Beeken, Suzanne H Richards

**Affiliations:** Leeds Institute of Health Sciences, University of Leeds, Leeds.; Leeds Institute of Health Sciences, University of Leeds, Leeds.; Exeter Collaboration for Academic Primary Care, College of Medicine and Health, University of Exeter, Exeter.; Leeds Institute of Health Sciences, University of Leeds, Leeds.; Leeds Institute of Health Sciences, University of Leeds, Leeds.; Leeds Institute of Health Sciences, University of Leeds, Leeds.

**Keywords:** general practice, physical activity, video recording

## Abstract

**Background:**

Clinical guidance recommends promoting physical activity during general practice consultations. The frequency and content of physical activity discussions in UK general practice are poorly understood.

**Aim:**

To explore the content of physical activity discussions during routine consultations between patients and GPs.

**Design and setting:**

Secondary analysis was undertaken of video-recorded UK general practice consultations from the One in a Million study, which was conducted in the West of England.

**Method:**

In total, 294 consultation transcripts were available; these were screened to identify consultations that included or omitted physical activity advice when recommended by National Institute for Health and Care Excellence guidance. The content, quality, and depth of advice provided by GPs were scored to ascertain how meaningful the advice was.

**Results:**

Physical activity was relevant to management according to clinical guidance in 175/294 (59.5%) consultations. In 64 (36.6%) of these consultations, physical activity was discussed as part of clinical management; the depth of discussion was judged as ‘meaningful’ in 22 (12.6%) consultations. Although physical activity advice tended to be given most often for musculoskeletal problems, depth of advice did not appear to be related to the presenting problem. When physical activity advice was relevant and omitted, consultations prioritised another overriding presenting problem, or clinical management focused on another intervention.

**Conclusion:**

Physical activity advice, following national guidance, was potentially relevant to more than half of GP consultations; GPs delivered advice of varying depth in a third of these consultations. Future work should explore ways of delivering physical activity advice effectively, efficiently, and equitably within the constraints of general practice.

## Introduction

The personal and population health consequences of physical inactivity on major non-communicable diseases are well documented.^1^ In the UK, more than a third of adults report low levels of physical activity^2^^,^^3^ and physical inactivity has been estimated to account for 17% of deaths,^3^ costing the economy £7.4 billion and the NHS £1 billion annually.^4^

Promoting positive health behaviours, including physical activity, is recommended across multiple National Institute for Health and Care Excellence (NICE) guidelines.^5^ NHS England’s Making Every Contact Count initiative encourages opportunistic discussions about physical activity in consultations across all NHS services.^6^ General practice has a key role in promoting physical activity as it accounts for 90% of NHS patient contacts, equating to ∼1 million consultations per day.^7^^,^^8^ However, there is evidence that GPs lack confidence and training in physical activity advice, and that time constraints are a major barrier to implementation.^9^^,^^10^ Qualitative studies highlight patient perceptions that conversations about physical activity during general practice consultations, if they occur at all, often lack substance.^10^ One analysis of audio-taped consultations^11^ found that 21% of patients could not accurately recall physical activity discussions with their GP because the advice was too brief or was delivered in a rushed manner.

There is little research on the frequency and content of physical activity advice given in general practice, which is needed to inform future improvement strategies.^12^ This study explored the content of physical activity discussions during routine consultations between patients and GPs. The authors examined whether physical activity discussions reflect NICE guideline recommendations, according to clinical presentations (Box 1 and Supplementary Table S1). They also explored the quality and depth of physical activity discussions, including whether the benefits of physical activity were mentioned, and whether advice was tailored to current levels of activity, health condition(s), and any disabilities, with signposting to local support.^13^

**Box 1.** Summary of *Physical Activity: Brief Advice for Adults in Primary Care* (NICE public health guideline PH44)^13^

In this NICE guideline, ‘brief advice’ means verbal advice, discussion, negotiation, or encouragement — with or without written or other support or follow-up. Furthermore, it states that such advice can vary from being basic to a more extended, individually focused discussion. The guideline defines physical activity as any bodily movement produced by skeletal muscles that requires energy expenditure, such as daily activity, active recreation, and sport. It further outlines that health-enhancing physical activity includes that which is cardiovascular, that which strengthens muscles and bones, and balance training types of activity. Benefits are highlighted, including the prevention and management of coronary heart disease, type 2 diabetes, stroke, mental health problems, musculoskeletal conditions, and some cancers, as well as the positive effect on wellbeing and mood, a sense of achievement, relaxation, and release from daily stress. Recommendation 1 focuses on identifying adults who are not currently meeting the UK physical activity guidelines. It suggests this could be done when the opportunity arises during a consultation with a primary care practitioner or as part of a planned session on management of long-term conditions. Recommendation 2 focuses on the delivery and follow-up of brief advice. It suggests that, when delivering brief advice, it is tailored to the person’s: motivations and goals; current level of activity and ability; circumstances, preferences, and barriers to being physically active; and health status (for example, whether they have a medical condition(s) or disability). It also suggests: providing information about local opportunities to be physically active, considering abilities, preferences, and needs; considering giving a written outline of the advice and goals that have been discussed; and recording the outcomes of discussions. Finally, it recommends follow-up when another appointment or opportunity could consist of a conversation about what physical activity has been engaged with, or what progress towards the patient’s goals has been attained, including achievement of the UK physical activity guidelines. Recommendations 3 to 5 are focused on: commissioning; systems to support brief advice, such as Read codes and information about local opportunities to be active; and the content and advice for information and training for primary care practitioners.

*NICE = National Institute for Health and Care Excellence.*

## Method

Video-recorded consultations are recommended for researching doctor–patient interactions.^14^^–^^16^ This cross-sectional study was a secondary analysis of video records held in the One in a Million dataset.^17^ The available dataset included 294 video records and verbatim transcripts of unselected primary care consultations with adults aged 18–96 years.^18^ Patients consulted with one of 23 GPs from 12 general practices serving areas of high and low deprivation in the West of England from July 2014 to April 2015. Participants consented for data to be reused for research purposes, subject to ethical approval. The videos and transcripts were supplemented with descriptive data on patients’ and GPs’ demographics, duration of consultations, and *International Classification of Primary Care, 2nd edition* coding of presenting problems. Each GP contributed consultations for between eight and 20 patients.

**Table:** How this fits in

Physical activity is below recommended levels in the UK population. National guidance recommends that general practice should routinely offer advice on physical activity. This analysis found that just over a third of eligible consultations included discussions about physical activity; although some of these discussions achieved a reasonable level of depth, most tended to be superficial. Future initiatives need to focus on how general practice can support physical activity more consistently and meaningfully within system constraints.

### Sampling

All 294 transcripts were screened by three assessors, who are GPs. Transcripts eligible for inclusion were identified in two stages. Initially, consultations with any mention of physical activity, either by the patient or GP, were identified. Relevant data were extracted into Microsoft Excel for analysis. The definition of physical activity included any mention of physical movement (for example, walking or gardening), exercise (planned bouts of physical activity for health benefits — for example, swimming or going to the gym), or sport (physical activity engagement in which rules are adhered to — for example, football).^19^^–^^21^ This preliminary sampling was intentionally broad to ensure consultations including any descriptions of physical movement were identified for further coding. The authors then identified and coded consultations eligible for the main analysis — that is, those in which physical activity was discussed as part of patient management in the context of the presenting consultation problem(s) and recommended by relevant NICE guidance.

The authors also included consultations in which opportunities to discuss or deliver advice for physical activity were potentially missed. These potential missed opportunities included consultations in which physical activity advice was relevant to the presenting problem and corresponding clinical guideline physical activity recommendation, but no advice was given. A coding framework based on NICE guidelines and Moving Medicine’s codesigned online physical activity consultation guides^22^^,^^23^ was used to judge when such missed opportunities occurred (Boxes 1 and 2, and Supplementary Table S1) — for example, when a patient consulting about hypertension received no advice on physical activity, as recommended by NICE guidance.^24^ Uncertainties were discussed and resolved between two authors who rated the consultations.

**Box 2.** Summary of Moving Medicine’s adult consultation guides

Moving Medicine is an online resource for clinicians and allied health professionals that provides evidence-based, condition-specific consultation guides on physical activity that can be used during consultations with patients. For each condition there is advice and information for 1-minute, 5-minute, and >5-minute consultations. The 1-minute conversation guides follow an ‘ask, explain, invite’ format, with direct links to research evidence that supports the advice provided. The guides for adult patients include, but are not limited to, the following conditions or circumstances: ischaemic heart disease, primary prevention, type 2 diabetes, obesity, anxiety, depression, musculoskeletal pain, inflammatory rheumatic disease, falls and frailty, pregnancy, postnatal, and cancer.

Within the sampling frame, the authors undertook a stratified random sample, using a random number generator (https://www.calculatorsoup.com/calculators/statistics/random-number-generator.php), selecting six consultations for each of the 23 GPs (*n* = 138) in the archive. Two authors then double-coded the content of the transcripts for these 138 selected consultations. Within this sample, two videos per GP (*n* = 46) were then purposively sampled to check that the coding of the transcripts captured sufficient detail to support analysis without a need to watch the associated videos. The authors purposively selected videos to capture a breadth of consultations, including any mention of physical activity or a potential missed opportunity. This quality-assurance step confirmed that the coding of the transcripts was sufficient.

### Consultation quality

The sub-sample of 46 videos was assessed for quality using the Global Consultation Rating Scale (GCRS).^25^^,^^26^ This observer-assessed instrument is designed to measure the effectiveness of communication and consultation process skills across a doctor–patient consultation. Two authors scored each video consultation on 12 overall domains (initiating the session, problem identification, problem exploration, patient’s perspective, non-verbal communication, developing rapport, providing structure, providing correct amount and type of information, aiding accurate recall and understanding, incorporating the patient’s perspective, planning and shared decision making, and closure).^25^ Each domain was scored as follows:

- 0 = not done or poor;
- 1 = adequate; or
- 2 = good.

A total score of 0–24 was derived, with higher scores representing better-quality consultations.

The authors contacted the original authors of the GCRS and received written guidance on how to apply the instrument. Two authors performed a series of independent coding and dual coding to ensure consistent use of the tool. The mean (standard deviation [SD]) quality scores for ‘any mention’ versus ‘missed opportunity’ consultations were visually inspected for any apparent differences; given the small sample size, no inferential testing was employed.

### Data analysis

The content of qualitative data from transcripts of consultations was coded by three authors using an iterative approach,^27^^,^^28^ with several cycles of coding and analysis. All coders were early-career academic GPs with varied experience of quantitative and qualitative research methods, medical education, and GP training. The coding strategy was discussed with the wider research team at monthly study meetings. Through this iterative process, the authors focused their main analysis on transcripts according to whether or not the physical activity advice offered was relevant to existing management guidelines for the clinical presentation (see Supplementary Table S1). For those consultations in which physical activity advice was relevant to clinical management, the depth of physical activity discussions was assessed. This was done according to:

- any mention of the importance of physical activity to the management of the presenting clinical problem(s);
- whether advice was tailored to account for the presenting problem or any relevant background medical conditions; and
- whether specific advice was given on how to improve physical activity within a management plan, or an onward referral was made for physical activity support.

The depth of physical activity discussions was categorised as:

- ‘meaningful’ if at least one of these three areas was addressed; or
- ‘superficial’ if none was addressed.

Consultations in which patients presented with multiple problems or those that involved medically complex cases were also explored.

For ‘missed opportunities’ the following were explored:

- whether the main focus of the consultation was irrelevant to physical activity; and
- whether consultations covered multiple or complex problems.

Content coding is presented using descriptive statistics illustrated with quotations from individual patients, individual GPs, or as dialogues between patients and GPs.

### Patient and public involvement and engagement

Patient and public involvement and engagement (PPIE) was incorporated in accordance with Guidance for Reporting Involvement of Patients and the Public, version 2, guidance.^29^ The study plan was initially developed in consultation with an established PPIE group that had experience of advising on implementation research in primary care.^30^ The group was not involved in participant recruitment as this was a secondary analysis. The authors subsequently engaged PPIE groups that included patients, carers, and retired healthcare workers with experience of cardiometabolic risk factors as well as musculoskeletal and long-term conditions, reflecting the clinical content of many of the consultations. Emerging results were shared during three online meetings and one face-to-face meeting, which contributed to the interpretation of the study findings.

## Results

### Sample characteristics

Of 294 patients in the total sample, 190 (64.6%) were female, 261 (88.8%) were of White ethnicity, and 116 (39.5%) were in the fourth and fifth most deprived population quintiles (Table 1). Thirteen (56.5%) GPs were female, nine (39.1%) were aged ≥51 years, all were White, and 13 (56.5%) had been qualified as GPs for ≥16 years (Table 2). One, two, or three problems were discussed in 155 (52.7%), 85 (28.9%), and 43 (14.6%) consultations, respectively, while 10 (3.4%) consultations covered ≥4 problems; data for one patient not recorded. The mean consultation duration was 11 mins 56 secs (range 1 min 19 secs–37 mins 54 secs).

**Table 1.** Sample characteristics of all patients (*n* = 294), and patients in consultations relevant to guideline-recommended physical activity advice (*n* = 175)

**Characteristic**	**All patients, *n* (%)**	**Patient consultations where physical activity advice relevant, *n* (%)**
**Sex**		
Male	103 (35.0)	60 (34.3)
Female	190 (64.6)	115 (65.7)
Not reported	1 (0.3)	

**Age, years**		
18–30	60 (20.4)	33 (18.9)
31–40	36 (12.2)	19 (10.9)
41–50	44 (15.0)	36 (20.6)
51–65	67 (22.8)	42 (24.0)
66–80	61 (20.7)	34 (19.4)
≥81	14 (4.8)	4 (2.3)
Not reported	12 (4.1)	7 (4.0)

**Ethnic group**		
Asian/Asian British	5 (1.7)	4 (2.3)
Black/African/Caribbean/Black British	12 (4.1)	5 (2.9)
Mixed/multiple ethnic groups	6 (2.0)	5 (2.9)
Other ethnic group	4 (1.4)	4 (2.3)
Not reported	6 (2.0)	3 (1.7)
White/White British/White other	261 (88.8)	154 (88.0)

**Deprivation quintile^a^**		
1 (least deprivation)	94 (32.0)	51 (29.1)
2	50 (17.0)	25 (14.3)
3	33 (11.2)	20 (11.4)
4	42 (14.3)	29 (16.6)
5 (greatest deprivation)	74 (25.2)	50 (28.6)

a *Data unavailable for one patient.*

**Table 2.** Sample characteristics of GPs (*n* = 23)

**Characteristic**	***n* (%)**
**Gender**	
Male	10 (43.5)
Female	13 (56.5)

**Age, years**	
31–40	7 (30.4)
41–50	7 (30.4)
≥51	9 (39.1)

**Ethnicity**	
White/White British	23 (100.0)

**Years since GP qualification**	
≤5	4 (17.4)
6–15	6 (26.1)
16–25	7 (30.4)
≥26	6 (26.1)

### Physical activity discussions

**Figure 1.**
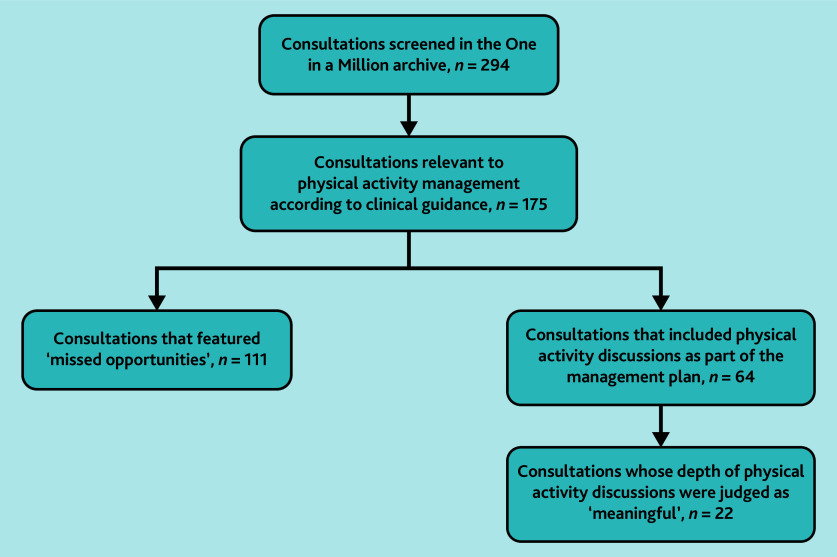
Transcript inclusion flowchart.

Physical activity advice was, or would have been, relevant to management according to clinical guidance in 175/294 (59.5%) consultations (Figure 1). Table 1 summarises the patient characteristics of this sub-group. Of these 175 consultations, 64 (36.6%) included physical activity discussions as part of the management plan (Figure 1), with a mean duration of 12 mins 42 secs (range 3 mins 26 secs–34 mins 18 secs). The depth of physical activity discussions was judged as ‘meaningful’ in 22/175 (12.6%) consultations and ‘superficial’ in 42/175 (24.0%) consultations.

The authors identified 111/175 (63.4%) consultations as representing ‘missed opportunities’ to discuss physical activity according to related clinical guidance (Figure 1), with a mean consultation duration of 13 mins 19 secs (range 1 min 19 secs–37 mins 54 secs). Examples of clinical presentations in which physical activity advice was not directly relevant included simple urinary tract infections and skin problems.

### Consultation quality

The mean GCRS score was 21.3 (SD 2.97; range 13–24). There were no apparent differences between overall quality scores and whether a consultation included any mention of physical activity (mean 21.4; SD 2.91) or a missed opportunity (mean 21.1; SD 3.08) (data not shown).

### Influence of the presenting problem

When it was given, physical activity advice was provided most frequently for musculoskeletal problems (40 consultations, 15 of which were considered meaningful), mental health problems (17 consultations, seven of which were considered meaningful), and cardiometabolic problems (11 consultations, three of which were considered meaningful). Cardiometabolic problems included conditions such as hypertension, obesity, and type 2 diabetes (data not shown). Mentions of physical activity included the following:

*‘I think generally it’s thought, it’s weight bearing as well during exercise is better. And swimming is good in terms of you getting your heart rate up, and so on. I’m not saying you have to go running or anything like that, but just a brisk walk, anything like that … because it just loads the joints a little bit. Do you see what I mean?’*

(GP, male)

GP: *‘Are you exercising?’*

Patient: *‘No, I don’t really have enough time.’*

GP: *‘Yes, it’s a struggle, isn’t it? Try and make time for it because it will improve your anxiety as well, ideally at least half an hour of brisk walking every day will dramatically improve things.’*

Patient: *‘I do that, I walk everywhere.’*

GP: *‘Good. Officially studies suggest three hours a week of cardiovascular exercise, where it’s actually making you sweat. So if you can achieve that, over the months you will just start to notice slowly a benefit from it.’* (GP: female; patient: female, aged 18–30 years)

Musculoskeletal problems sometimes included signposting or referral to other professionals, such as physiotherapists, for specific exercises as part of a management strategy:

*‘What we could do to help that would be to get you to see either the physio or the chiropractor — we can talk about the differences between those — who would help you to strengthen up your back.’*

(GP, female)

There were some examples of GPs advising on moderating activity, because to a limitation, or avoiding activity all together:

*‘I would not swim until you’ve seen the musculoskeletal people because swimming for the shoulders can be … if you got a tear.’*

(GP, female)

For cardiometabolic problems, physical activity advice was sometimes given as part of management for multiple modifiable risk factors:

*‘A lot of people do have high blood pressure that need*[s] *medication for life, but if there’s a recognisable thing that we think is causing it and you’re at that borderline level, then often making lifestyle changes, looking at your exercise, looking at your diet, looking at your salt intake, looking at your alcohol intake, all of these things can make a huge difference.’*

(GP, male)

Furthermore, there were examples of missed cues when a patient offered opportunities for the GP to provide support:

GP: *‘Because the thing is that I suspect that what that is doing is just reducing the very borderline hypertension, which is obviously having an impact on your renal function.’*

Patient: *‘This is it, I’m not really keen on taking another drug at all, but it’s just that this is ridiculous.’* (GP: female; patient: female, aged 66–80 years)

In consultations in which physical activity was relevant but not discussed, the focus of the consultation was often another overriding presenting problem that was not relevant to physical activity — for example, a consultation for cellulitis that also included a discussion about hypertension. Physical activity advice also tended to be omitted when clinical management focused on other interventions, such as encouraging a patient to engage in counselling for a mental health presentation or medication titration for hypertension.

### Multiple problems and medical complexity

Patients often presented with multiple problems — such as musculoskeletal, mental health, and cardiometabolic problems — at least one of which could justify physical activity advice. The presentation of multiple clinical problems did not seem to influence whether, or how, physical activity was discussed, with the average number of problems being approximately two in all scenarios. During one consultation, the GP gave a limited response to cues to discuss guideline-recommended physical activity in a patient with a background of rheumatoid arthritis, depression, lipid disorder, and elevated blood pressure:

Patient: *‘When I’m feeling well I don’t sit around, I am active. I go up in the garden … I try not to set my goals too high. So I do exercise, not so much walking, but I try to bend up and down.’*

GP: *‘Yes. I think that is all good. Again, I’m not sure how much that alone will lower the blood pressure by itself … we need to get your blood test done, we can check your cholesterol … given how the blood pressure’s been … might be a longer-term issue.’*

Patient: *‘I was going to try and do a little bit of swimming, but I must admit I haven’t really stuck to that. I know I should probably do a bit more exercise … And I would like to lose some weight, but I don’t know how you’d do that when — my appetite is huge sometimes … that is one of the drawbacks with the prednisolone.’*

GP: *‘Yes, yes. I don’t think there’s an easy way around that … Anything that you can make, a small sustainable change.’* (GP: male; patient: male, aged 51–65 years)

## Discussion

### Summary

It was observed that the provision of advice on physical activity, in accordance with national guidance, applied to more than half of patient consultations with GPs. Just over a third of these eligible consultations included discussions about physical activity, with meaningful discussions taking place in 12.6% of relevant consultations. Although physical activity advice tended to be given most often for musculoskeletal problems, no strong evidence that depth of advice was related to the presenting problem was found. No notable differences were observed in physical activity advice according to whether consultations covered multiple or complex problems, but other guideline-recommended aspects of care, such as prescribing, sometimes took priority.

### Strengths and limitations

To the best of the authors’ knowledge, this is the first study to observe adherence to national guidance on physical activity advice in UK general practice consultations. The use of video-recorded consultations with verbatim transcripts avoids recall bias that might otherwise be encountered with interviewees, and provides a level of detail that is unavailable through casenote reviews.^14^^–^^16^ Participants in the naturalistic One in a Million study^18^ were unaware that their consultations would be analysed specifically about physical activity, reducing the likelihood of behaviour change in response to study participation.

As this study was cross-sectional, it is not known what physical activity discussions may have taken place between patients and GPs before, or after, the index consultation. Furthermore, this study does not reflect consultations with other general practice staff, such as nurses and allied health professionals. This could lead to an underestimation of physical activity advice given in general practice, especially for long-term conditions requiring multiple consultations or instances when there was interpersonal continuity of care.

The assessment of adherence to NICE guidance involved a degree of judgement. Double coding was used and criteria were applied when assessing adherence to compensate for the authors’ own biases. However, there are some limitations of which it is important to be aware. The observed consultations took place from 2014–2015 and, as such, may not fully reflect the contemporary context, including evolving clinical guidance and increasing emphasis on physical activity promotion for general practice. In addition, the COVID-19 pandemic has changed practice and increased demand, with ongoing competing pressures and long-term staff shortages.^31^ Further to this, the recognised lack of ethnic diversity in this sample reduces generalisability to diverse populations that may benefit the most from physical activity support.^32^

Familiarity with guidance on physical activity is associated with a greater likelihood of raising physical activity in a consultation.^9^ Initiatives like the Physical Activity Clinical Champions Programme aim to provide clinician-to-clinician training on promoting physical activity in practice.^33^ It is unclear what previous physical activity counselling training the GPs in this sample received, and any likely impact on behaviour. Clinicians’ own physical activity levels can influence the frequency of physical activity discussions with patients;^34^ although some GPs referred to their own activity in the consultations, engagement in physical activity across the sample of GPs included in this analysis was not formally reported. A rigorous assessment of behavioural change techniques employed in consultations was beyond the scope of this study and, given the overall brevity of the physical activity discussions, unlikely to yield detailed insights.^35^ Although these are recognised limitations of the data available, the One in a Million dataset provides an exploratory snapshot of what physical activity advice is offered during general practice consultations in England.

### Comparison with existing literature

A systematic review of physical activity brief interventions^10^ found that the proportions of primary care health professionals who reported delivering physical activity advice ranged widely from 0.6% to 100%. Levels of patient-reported receipt of physical activity advice, ranging from 18% to 35%, are consistent with the observations of the study reported here. The review also highlighted that patients perceived that physical activity discussions often did not take place or lacked substance;^10^ this is consistent with the finding presented here that only 12.6% of consultations included sufficiently detailed advice that was tailored to individuals. An analysis of audio-taped consultations in an academic US primary care centre found that physical activity was mentioned in 72% of consultations,^11^^,^^36^^,^^37^ but that advice was often superficially presented — such as, ‘try to exercise’.

Health professional and patient factors may explain variations in the provision of physical activity advice. Health professionals who are female, report higher levels of physical activity themselves, or hold more positive beliefs about their capabilities are more likely to deliver physical activity advice than those who do not.^10^ Patient factors associated with the receipt of physical activity advice include high body mass index, being physically inactive, being in relatively poor health, and having higher consultation rates.^10^ Time constraints have consistently been reported as a major barrier to discussions about physical activity;^9^^,^^10^^,^^34^ this is unsurprising considering that multiple problems and their associated tasks need to be dealt with in a 10–12-minute timeframe. GPs may tend to prioritise other tasks, such as prescribing, over giving physical activity advice.^38^

### Implications for research and practice

The World Health Organization’s 2022 global status report on physical activity outlines that, since the adoption of the Global Action Plan on Physical Activity 5 years prior to the report, globally there has been little progress towards increasing population levels of physical activity.^39^ Survey data also indicate that physical inactivity among adults in high-income countries is double that of low-income countries,^40^ with high-income countries also set to incur the largest economic cost.^39^ Without effective action, physical inactivity will account for a predicted 500 million new cases of preventable major non-communicable diseases by 2030.^41^ The overwhelming majority (98.9%) of UK GPs responding to one survey^34^ believed that physical activity was important; however, the study presented here raises questions about the role and feasibility of general practice in delivering physical activity advice as recommended by national guidance.

A meta-analysis of 46 randomised trials^42^ found that physical activity interventions delivered, or prompted, by health professionals in primary care increased moderate-to-vigorous physical activity (MVPA) by a mean of 14.4 mins per week and increased the proportion of participants meeting MVPA guidelines by 33%. As MVPA has an inverse dose–response relationship with all-cause mortality, even modest increases in physical activity may translate into important population impacts;^43^ this justifies attempts to improve patient engagement in physical activity in general practice.

Clearly, not all consultations can include discussions about physical activity every time, even when these are clinically relevant and recommended by national guidance. Johansson *et al*^44^ estimated that faithful implementation of NICE guidance on brief physical activity, including the 1-minute screening for eligibility and 10-minute intervention delivery, would require 167 hours annually for a typical GP — equivalent to 15% of face-to-face time with patients. Given the growing burdens and competing priorities facing general practice, this would be neither feasible nor acceptable in the foreseeable future.^45^ This creates a dilemma for policy and practice, which currently risk widening, rather than closing, inequality gaps. As an example, patients with multiple morbidities and complex problems, who need longer consultations, probably stand to gain most from physical activity support but are less likely to receive it compared with those with less complexity and multimorbidity who require shorter consultation times.^32^ Research is needed to identify ways to integrate support for physical activity into general practice more effectively, efficiently, and equitably than presently occurs.

There are opportunities to build on emerging evidence about novel approaches to encourage people to be more physically active, such as Snacktivity™, which aims to encourage people to do small 2–5-min bouts of physical activity ‘snacks’ throughout the day.^46^^,^^47^ Other initiatives, such as parkrun practice, Couch to 5k, social prescribing, and link workers all offer signposting opportunities for general practice to promote physical activity. Future research into effective initiatives should involve health professionals and patients in design processes to ensure acceptability in practice and inclusion of underserved populations.^48^^,^^49^

## Data

This study used data from the One in a Million study. The One in a Million study dataset (https://doi.org/10.5523/bris.l3sq4s0w66ln1x20sye7s47wv) is controlled, due to participants potentially being identifiable. For more information on accessing the data, please visit: https://www.bristol.ac.uk/primaryhealthcare/researchthemes/one-in-a-million/accessing-the-data.

## References

[1] Lee I-M, Shiroma EJ, Lobelo F (2012). Effect of physical inactivity on major non-communicable diseases worldwide: an analysis of burden of disease and life expectancy. Lancet.

[2] British Heart Foundation (2017). Physical inactivity and sedentary behaviour report 2017.

[3] Office for Health Improvement and Disparities (2022). Physical activity: applying All Our Health.

[4] National Institute for Health and Care Excellence (2018). Physical activity and the environment NG90.

[5] National Institute for Health and Care Excellence (2022). Clinical knowledge summaries.

[6] Public Health England (2016). Making Every Contact Count (MECC): consensus statement.

[7] Gibbons DC, Bindman AB, Soljak MA (2012). Defining primary care sensitive conditions: a necessity for effective primary care delivery?. J R Soc Med.

[8] Brooks J, Ahmad I, Easton G (2016). Promoting physical activity: the general practice agenda. Br J Gen Pract.

[9] Chatterjee R, Chapman T, Brannan MGT, Varney J (2017). GPs’ knowledge, use, and confidence in national physical activity and health guidelines and tools: a questionnaire-based survey of general practice in England. Br J Gen Pract.

[10] Hall LH, Thorneloe R, Rodriguez-Lopez R (2022). Delivering brief physical activity interventions in primary care: a systematic review. Br J Gen Pract.

[11] Bardach SH, Schoenberg NE, Howell BM (2017). Older patients’ recall of lifestyle discussions in primary care. J Appl Gerontol.

[12] Sturgiss E, van Boven K (2018). Datasets collected in general practice: an international comparison using the example of obesity. Aust Health Rev.

[13] National Institute for Health and Care Excellence (2013). Physical activity: brief advice for adults in primary care PH44.

[14] Coleman T (2000). Using video-recorded consultations for research in primary care: advantages and limitations. Fam Pract.

[15] Salisbury C, Procter S, Stewart K (2013). The content of general practice consultations: cross-sectional study based on video recordings. Br J Gen Pract.

[16] Parry R, Pino M, Faull C, Feathers L (2016). Acceptability and design of video-based research on healthcare communication: evidence and recommendations. Patient Educ Couns.

[17] Barnes R, Ridd MJ, Jepson MJ, Salisbury CJ (2017). One in a Million: a study of primary care consultations.

[18] Jepson M, Salisbury C, Ridd MJ (2017). The ‘One in a Million’ study: creating a database of UK primary care consultations. Br J Gen Pract.

[19] Caspersen CJ, Powell KE, Christenson GM (1985). Physical activity, exercise, and physical fitness: definitions and distinctions for health-related research. Public Health Rep.

[20] World Health Organization (2024). Physical activity.

[21] Sancassiani F, Machado S, Preti A (2018). Physical activity, exercise and sport programs as effective therapeutic tools in psychosocial rehabilitation. Clin Pract Epidemiol Ment Health.

[22] Reid H, Caterson J, Smith R (2022). What do healthcare professionals want from a resource to support person-centred conversations on physical activity? A mixed-methods, user-centric approach to developing educational resources. BMJ Open Sport Exerc Med.

[23] Moving Medicine. Prescribing movement for adults. https://movingmedicine.ac.uk/consultation-guides/for-adults.

[24] National Institute for Health and Care Excellence (2023). Hypertension Scenario: management.

[25] Burt J, Abel G, Elmore N (2018). Rating communication in GP consultations: the association between ratings made by patients and trained clinical raters. Med Care Res Rev.

[26] Burt J, Abel G, Elmore N (2014). Assessing communication quality of consultations in primary care: initial reliability of the Global Consultation Rating Scale, based on the Calgary–Cambridge Guide to the Medical Interview. BMJ Open.

[27] Hsieh H-F, Shannon SE (2005). Three approaches to qualitative content analysis. Qual Health Res.

[28] Green J, Thorogood N (2013). Qualitative methods for health research.

[29] Staniszewska S, Brett J, Simera I (2017). GRIPP2 reporting checklists: tools to improve reporting of patient and public involvement in research. BMJ.

[30] Gray-Burrows KA, Willis TA, Foy R (2018). Role of patient and public involvement in implementation research: a consensus study. BMJ Qual Saf.

[31] Burn E, Fisher R, Locock L, Smith J (2022). A longitudinal qualitative study of the UK general practice workforce experience of COVID-19. Prim Health Care Res Dev.

[32] Public Health England (2021). Understanding and addressing inequalities in physical activity: evidence-based guidance for commissioners.

[33] Carlin L, Musson H, Adams E (2019). Moving Healthcare Professionals Programme Phase 1 Clinical Champions’ Physical Activity Training Programme: final evaluation report. OSF Preprints.

[34] Lowe A, Myers A, Quirk H (2022). Physical activity promotion by GPs: a cross-sectional survey in England. BJGP Open.

[35] Ayre E, Lee JJ, Frie K (2023). GP delivered brief weight loss advice: associations between in-consultation behaviour change techniques and patient weight loss in recorded primary care discussions. Health Psychol Behav Med.

[36] Bardach SH, Schoenberg NE (2014). The content of diet and physical activity consultations with older adults in primary care. Patient Educ Couns.

[37] Bardach SH, Schoenberg NE (2018). The role of primary care providers in encouraging older patients to change their lifestyle behaviors. Clin Gerontol.

[38] Woodhead G, Sivaramakrishnan D, Baker G (2023). Promoting physical activity to patients: a scoping review of the perceptions of doctors in the United Kingdom. Syst Rev.

[39] World Health Organization (2022). Global status report on physical activity 2022.

[40] Guthold R, Stevens GA, Riley LM, Bull FC (2018). Worldwide trends in insufficient physical activity from 2001 to 2016: a pooled analysis of 358 population-based surveys with 1.9 million participants. Lancet Glob Health.

[41] Santos AC, Willumsen J, Meheus F (2023). The cost of inaction on physical inactivity to public health-care systems: a population-attributable fraction analysis. Lancet Glob Health.

[42] Kettle VE, Madigan CD, Coombe A (2022). Effectiveness of physical activity interventions delivered or prompted by health professionals in primary care settings: systematic review and meta-analysis of randomised controlled trials. BMJ.

[43] Arem H, Moore SC, Patel A (2015). Leisure time physical activity and mortality: a detailed pooled analysis of the dose–response relationship. JAMA Intern Med.

[44] Johansson M, Guyatt G, Montori V (2023). Guidelines should consider clinicians’ time needed to treat. BMJ.

[45] Lawson E (2023). The global primary care crisis. Br J Gen Pract.

[46] Gokal K, Amos-Hirst R, Moakes CA (2022). Views of the public about Snacktivity^™^: a small changes approach to promoting physical activity and reducing sedentary behaviour. BMC Public Health.

[47] Sanders JP, Biddle SJH, Gokal K (2021). ‘Snacktivity^™^’ to increase physical activity: time to try something different?. Prev Med.

[48] INVOLVE (2019). Co-production in action: number two.

[49] National Institute for Health and Care Research (2024). Improving inclusion of under-served groups in clinical research: guidance from INCLUDE project. https://www.nihr.ac.uk/documents/improving-inclusion-of-under-served-groups-in-clinical-research-guidance-from-include-project/25435.

